# Development and demonstration of end‐to‐end testing for intra‐fraction motion‐managed workflows

**DOI:** 10.1002/mp.18042

**Published:** 2025-08-24

**Authors:** Stijn Oolbekkink, Jochem W. H. Wolthaus, Bram van Asselen, Peter R. S. Stijnman, Bas W. Raaymakers

**Affiliations:** ^1^ Department of Radiotherapy University Medical Center Utrecht Utrecht the Netherlands

**Keywords:** end‐to‐end testing, intra‐fraction motion‐management, MRI guided radiotherapy

## Abstract

**Background:**

Intra‐fraction motion management techniques, including beam gating and intra‐fraction drift correction (IDC), have recently been introduced on the Unity MR‐linac (Elekta AB, Stockholm, Sweden) to mitigate the dosimetric impact of motion during treatment. However, residual motion (e.g., within the gating window) still affects the delivered dose, causing deviations from the statically planned dose. Conventional end‐to‐end (E2E) testing does not incorporate such (known) motion, hampering evaluation of motion managed workflows.

**Purpose:**

This study develops and demonstrates novel methods that incorporate known motion before treatment delivery. Using such a reference dose distribution allows for E2E testing of intra‐fraction motion‐managed workflows.

**Methods:**

A novel approach was developed to assess the E2E accuracy for motion‐managed delivery techniques by comparing the measured dose distribution to a reference dose distribution that incorporates the applied motion during the delivery. Two motion‐included reference dose distributions were generated and evaluated: (1) A Priori Motion‐Included (APriMI) dose distribution which uses the known (periodic) motion to estimate the influence of anatomical motion on the dose distribution, and incorporates this into a new dose distribution; and (2) the Posteriori Motion‐Included (PostMI) dose distribution, which adds an external trigger to relate the beam‐on/off time to the motion of the setup. This allows for evaluation of non‐periodic motion, or a drift motion during IDC workflows. In addition to these, the conventionally used static treatment planning system (TPS) dose distribution was used as a reference dose distribution.

Several scenarios were evaluated: static (no phantom motion), two unmanaged, and two motion‐managed scenarios using the Comprehensive Motion Management (CMM) software (Elekta AB, Stockholm, Sweden) for gated and IDC workflows, with $\cos^{4}$ and linear drift motion patterns. All measurements were performed on a clinical Unity MR‐linac equipped with CMM software, using film dosimeters for high spatial resolution dose distribution assessment. The geometric and dosimetric E2E accuracy of the workflow were evaluated for all scenarios.

**Results:**

First, the static benchmark scenario was evaluated and showed high agreement between the measured dose distribution and all reference dose distributions (i.e., static, APriMI, and PostMI). For the motion‐included scenarios, excellent agreement was observed between the measured and calculated dose distributions in both unmanaged and managed cases when using either APriMI or PostMI. The largest geometric shift in the motion included scenarios was 0.3 mm, comparable to the static scenario. Dosimetric accuracy, evaluated using a global gamma index (2%/2 mm), exceeded 95.5%. As expected, larger deviations occurred when the static dose distribution was used as a reference, with geometric shifts up to 9.0 mm and gamma pass rates as low as 17.8%.

**Conclusions:**

E2E testing of intra‐fraction motion‐managed workflows is possible using APriMI and PostMI dose distributions. Strong agreement was observed with these motion‐included distributions, while larger deviations were seen with the static dose distribution. These findings highlight the need for reference dose files that account for actual motion in the measurement setup to assess E2E accuracy of motion‐included workflows.

## 1 INTRODUCTION

The accuracy of the complete radiotherapy workflow is determined with an end‐to‐end (E2E) test, which starts with the acquisition of pre‐treatment planning images, and ends with dose delivery and the analysis of the delivered dose distribution.^1^, ^2^, ^3^ The delivered dose distribution, measured by a dosimeter, is compared to the planned (reference) dose distribution. In doing so, an overall geometrical and dosimetric E2E accuracy of the delivered dose distribution relative to the intended reference dose distribution is obtained for the treatment.

The MR‐linac, the combination of an MRI scanner and a linear accelerator (linac), is used to deliver adaptive radiotherapy treatments.^4^, ^5^, ^6^, ^7^ By using MR images acquired before dose delivery, adaptive treatment plans can be created to account for day‐to‐day anatomical changes.^8^ In studies by Bernchou et al. and Oolbekkink et al. assessing the E2E accuracy of the Unity MR‐linac (Elekta AB, Stockholm, Sweden), the static (i.e., no intra‐fraction motion) online workflows demonstrated high geometric and dosimetric accuracy.^9^, ^10^ Oolbekkink et al. reported that without intra‐fraction motion the 3D geometrical accuracy was equal to or better than 0.3 mm, determined using 3D gel dosimeters.

Recently, intra‐fraction motion‐managed workflows have been introduced on the Unity MR‐linac, enabling tracking and management of motion.^11^ There are two types of intra‐fraction motion‐managed workflows: beam gating for respiratory motion, and intra‐fraction drift correction (IDC) for slower drift motions. The latter involves generating a new adapt‐to‐position (ATP) treatment plan based on the original online plan and the updated target position resulting from the drift, typically within 1 to 2 min. In this IDC plan, the shift of the target is accounted for using segment aperture morphing (SAM).

Intra‐fraction motion‐managed workflows inherently involve motion during beam delivery, thereby influencing the deposited dose distribution (e.g., motion within gating window or drift motion), making it different from the static reference dose.^12^, ^13^ However, conventional static E2E tests do not account for any intra‐fraction motion, limiting their ability to evaluate dynamic workflows.^2^ As a result, the effects of latencies, motion prediction errors, and new IDC treatment plans on the delivered dose distribution are overshadowed by motion induced effects. These uncertainties affect the result of the workflow, and must be included.

Determining the E2E accuracy of such workflows is not trivial, as the delivered dose distribution deviates from the planned dose distribution. However, when the motion is known, it can be incorporated into the calculated dose distribution, resulting in a reference dose distribution that includes motion effects. This enables a proper determination of the overall E2E accuracy of intra‐fraction motion‐managed treatment workflows, including the measurement setups with motion.

In this study, we present and assess a novel method in which a motion‐included reference dose distribution is used to compare to the measured dose distribution, enabling determination of the E2E accuracy of intra‐fraction motion‐managed workflows. The study proposes two different methods for calculating the reference dose distribution, aiming to more accurately identify true machine‐ or motion management‐related errors by accounting for the known dose distribution changes caused by known motion.

## 2 MATERIALS AND METHODS

This study investigated three types of reference dose distributions for E2E testing of intra‐fraction motion‐managed workflows by evaluating their dosimetric and geometric accuracy using film dosimeter measurements. The Comprehensive Motion Management (CMM) software (Elekta AB, Stockholm, Sweden) was used for gated and IDC workflows on the Unity MR‐linac for intra‐fraction motion management.^11^ Throughout this study, the IEC 61217 coordinate system is used.^14^

### 2.1 Three reference dose distributions

#### 2.1.1 Static dose distribution

The first reference dose distribution was the static planned dose, which is the standard result of the optimization process in the Monaco (v. 6.2.1.0, Elekta AB, Stockholm, Sweden) treatment planning system (TPS) using the GPU‐oriented Monte Carlo Dose (GPUMCD) (Elekta AB, Stockholm, Sweden) engine for dose calculations.^15^, ^16^ Notably, this dose distribution does not account for intra‐fraction motion. The dose distribution had a voxel size of 1.0 × 1.0 × 1.0 ${\mathrm{mm}}^{3}$ and a statistical uncertainty of 0.5% per segment in the dose calculation.

#### 2.1.2 APriMI dose distribution

**FIGURE 1:**
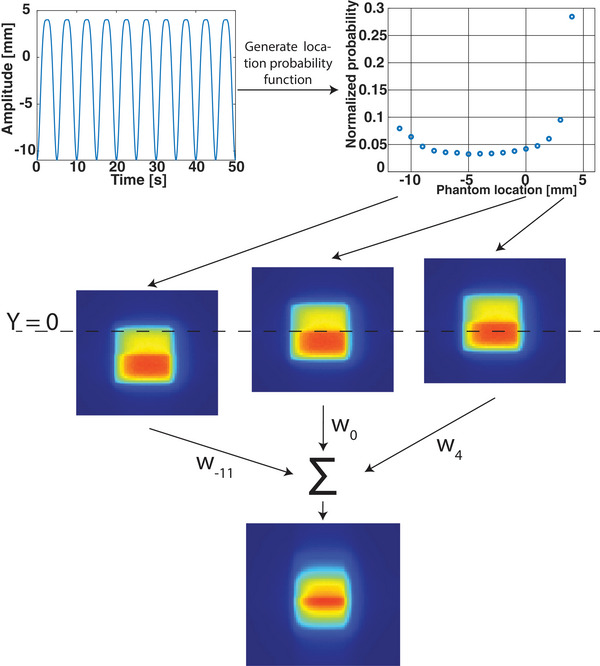
Schematic overview of the process for obtaining the motion‐included dose distribution. A normalized location probability density function is generated from the motion. For each discrete position, a dose distribution is calculated using the shifted anatomy. The final dose distribution is then obtained by summing the weighted dose distributions, where $W_{n}$ represents the weight corresponding to location *n* of the motion.

The dosimetric impact of anatomical motion on the dose distribution can be assessed by recalculating and adding multiple shifted anatomical states, similar to amplitude binning in 4DCTs.^17^ This method accounts for any inhomogeneities in the anatomy that influence the dose distribution, which would not be the case when applying a convolution‐based method to a single dose distribution.^12^, ^13^ A schematic overview of the generation of the motion‐included dose file is shown in Figure 1. The motion shifts were simulated in discrete 1 mm steps by adjusting the treatment plan isocenter, and the dose for each segment was recalculated using the GPUMCD dose engine for every shifted position,^15^, ^16^ leading to multiple dose distributions, each corresponding to a shifted anatomy. Each dose distribution had a voxel size of 1.0 × 1.0 × 1.0 ${\mathrm{mm}}^{3}$ and a statistical uncertainty per segment calculation in the dose calculation of 0.5% was used. Using the known motion information, a normalized position probability distribution was generated and applied to the shifted dose distributions. The sum of these position probability distributions was normalized to 1, and the corresponding dose distributions were weighted accordingly, subsequently resampled onto a common grid and accumulated, resulting in an accumulated motion‐included dose distribution with a voxel size of 1.0 × 1.0 × 1.0 ${\mathrm{mm}}^{3}$. Since known motion is used to determine the position probability distribution, this method assumes periodic motion. It also assumes that the duration of segment delivery is long compared to the motion cycle. During the scenario involving beam gating, the constraint to the CMM (i.e., the gating envelope) was also incorporated into the a Priori Motion‐Included Dose (APriMI) dose distribution, thereby reconstructing the dose distribution that should have been delivered.

#### 2.1.3 PostMI dose distribution

If the assumptions of APriMI are not valid, such as in cases of non‐periodic motion or when accounting for low monitor unit (MU) segments which violate the assumption of the APriMI, an external trigger is required to synchronize motion timing with dose delivery (beam on and off timings). Also, if non‐periodic drift motion is used, more information is required. This approach enables E2E testing of IDC workflows by providing information on when IDC adaptations are executed, the time required for IDC replanning, and the remaining MU to be delivered. Furthermore, it allows for the consideration of interplay effects during dose delivery. This method is referred to as the Posteriori Motion‐Included (PostMI) dose distribution.

Similar to the APriMI method, the PostMI method generates the motion‐included dose distribution by shifting the anatomy according to a known motion pattern. The PostMI however adds the time‐resolved external beam‐on trigger to the position probability distribution. The beam‐on trigger signal can be an independent time‐resolved dose detector, however, for the sake of simplicity in this proof of concept study, it was derived from the treatment record file (TRF), which stores linac parameters (such as the delivered MU) over time. Beam‐on time is defined based on time stamps where MU counter increments are non‐zero. During this study, the phantom's motion was recorded at 25 ms sampling intervals, with time stamps synchronized to Unity's time server.

### 2.2 Phantom setup

**FIGURE 2:**
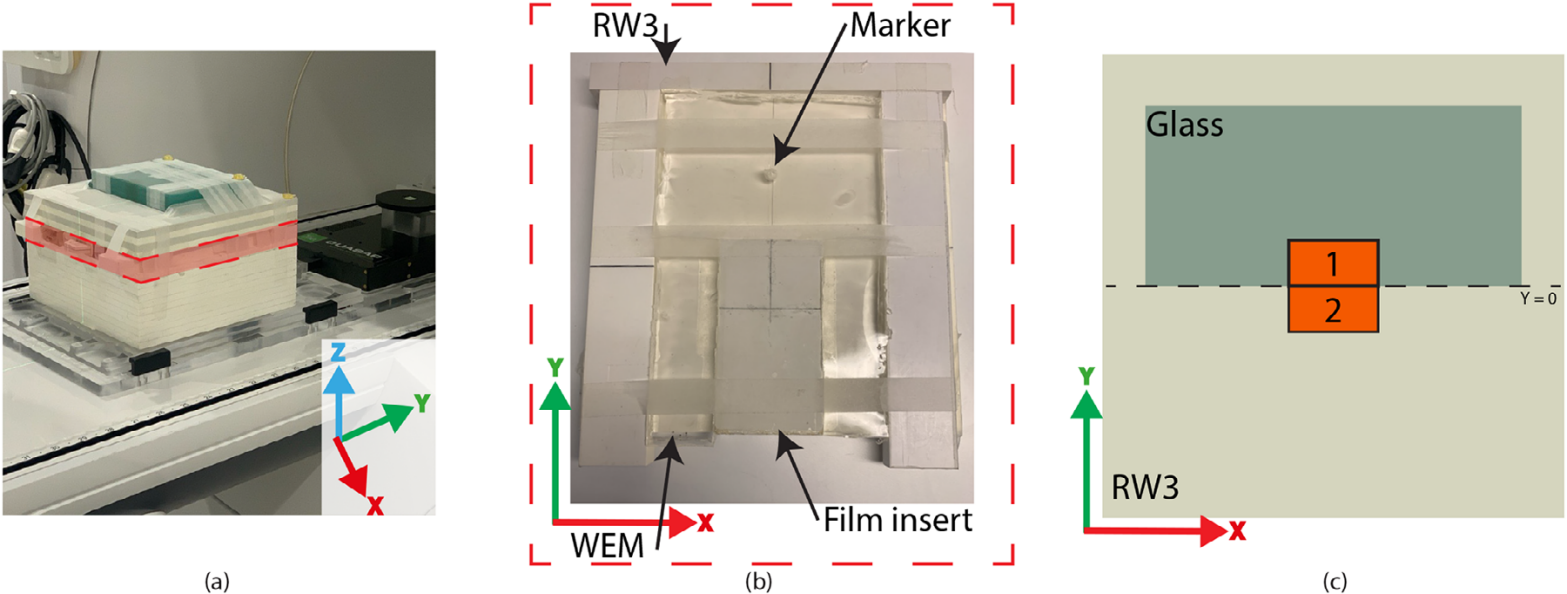
(a) An image of the full measurement setup, with the red box indicating the region shown in (b); (b) Close‐up of the highlighted region, displaying the RW3 material, marker used for tracking, WEM, and film insert; (c) Schematic top view of the measurement setup, showing the relative location of the two beam segments (1 and 2) within the phantom. Axes indicate the coordinate system used throughout this study. The IEC 61217 coordinate system is used. WEM, water‐equivalent material.

To experimentally assess the three reference dose distributions for E2E testing and to demonstrate the (potentially large) impact of motion on the measured dose distribution, a phantom with simple geometric features yet varying electron density (ED) was required. This allowed the simulation of large density differences (e.g., near the lung) and their impact on dose distribution during intra‐fraction motion. The phantom also needed to contain sufficient MR‐visible material for scanning and tracking. To meet these requirements, the custom measurement setup was designed using Clearsight Bolus water‐equivalent material (WEM) (Cortex Manufacturing LLC, Sororento, FL, USA), RW3 plates, glass, a zirconium marker, an insert, and film cassette with MR‐visible markers.^9^ The setup, shown in Figure 2a,b, was specifically designed to avoid small irregularities in the beam attenuation and scatter conditions. Therefore, 5 cm of RW3 was placed above the film dosimeter to ensure consistent scatter conditions, and a 4 cm thick glass slab was added over half of the planned high‐dose region to introduce a deliberate inhomogeneity. This glass creates a sharp, well defined change in ED within the measurement setup. The phantom was positioned on the Quasar Motion MR Platform (IBA QUASAR, Modus Medical Devices Inc., London, Ontario, Canada) to apply remotely controlled linear movement to the whole phantom assembly shown in Figure 2a along the *y*‐axis following pre‐programmed motion traces.^18^

### 2.3 Workflow

A treatment on the MR‐linac consists of two parts: the pre‐treatment workflow and the online workflow. Since a new treatment plan is made for every online workflow, E2E testing is only performed on the online workflow.

#### 2.3.1 Pre‐treatment planning

To highlight the effectiveness of, and the need for, the APriMI and PostMI methods, a simple two‐segment step and shoot treatment plan was created to evaluate the developed methods. The contour delineations and pre‐treatment plans were generated using the Monaco TPS on CT images (1.0 × 1.0 × 1.0 ${\mathrm{mm}}^{3}$) of the measurement setup, acquired with a Big Bore CT (Philips Healthcare, Best, the Netherlands). The RW3 plates were assigned an ED of 1.024, the motion platform an ED of 1.159, and the glass an ED of 2.000. An artificial box‐shaped gross tumor volume (GTV) of 30 × 30 × 10 ${\mathrm{mm}}^{3}$ was defined at the center of the film insert, and an isometric planning target volume (PTV) margin of 5 mm was applied around the GTV, serving as the gating envelope. During CMM‐managed workflows, dose is allowed to be delivered while the GTV remains within the gating envelope. The zirconium marker in the WEM, located approximately 5 cm from the top of the film cassette, was contoured and designated as a tracking structure for cine MR imaging. This marker position ensures no undesired beam scattering at the GTV location. To maximize the effects of target motion on the delivered dose, two abutting segments were created, covering a combined field size of 3.0 × 3.0 ${\mathrm{cm}}^{2}$ at isocenter (see Figure 2c).

The abutting segments reveal the impact of intra‐fraction motion on the dose distribution, where some regions may experience underdosage and others overdosage. Segment 1 was [$x_{1}$, $x_{2}$, $y_{1}$, $y_{2}$] = [1.5, −1.5, 1.5, 0] cm, while segment 2 was [$x_{1}$, $x_{2}$, $y_{1}$, $y_{2}$] = [1.5, −1.5, 0, −1.5] cm. Both segments were delivered from gantry angle 0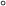, and each delivered 1500 MU. This resulted in maximum doses at the film dosimeter location of 8.9 Gy for segment 1 and 11.5 Gy for segment 2.

#### 2.3.2 Online workflow

To initiate the online MR‐linac workflow, MR images (T2‐weighted, acquired pixel size = 1.3 × 1.3 ${\mathrm{mm}}^{2}$, slice thickness = 1.0 mm) of the measurement setup were acquired at the reference position (*Y* = 0, Figure 2c). After image acquisition, the online MRI and pre‐treatment CT were registered. Manual adjustments to the registration were made, if necessary, by visually aligning the MR‐visible markers in the MRI with those on the pre‐treatment CT. Following registration, contours were propagated to the online MRI. All contours were propagated rigidly. If necessary, contours were adjusted to match the phantom setup.

The adapt‐to‐shape (ATS) strategy with adapt‐segments was chosen to create the online treatment plan for the measurements, which compensates for phantom shifts by moving the original segments.^8^ While the ATS method generally aims to reproduce the dose distribution of the reference plan, the use of adapt‐segments specifically restricts this to rigid shifts of the original segments based on the image registration vector. The online dose was calculated with a voxel size of 3.0 × 3.0 × 3.0 ${\mathrm{mm}}^{3}$ and a 3% statistical uncertainty per segment. To compare with the measured data, the treatment plan was recalculated post‐treatment using a voxel size of 1.0 × 1.0 × 1.0 ${\mathrm{mm}}^{3}$ and a statistical uncertainty of 0.5% per segment. This recalculation aimed to minimize discretization errors and reduce statistical uncertainty in the dose calculation. The recalculated dose distribution serves as the reference for the static dose distribution method (see Section 2.1.1).

Monitoring of 3D target motion using CMM was performed using 2D interleaved sagittal and coronal cine images. To capture respiratory motion, a 6 Hz (3 Hz per imaging plane) balanced turbo field echo (acquired pixel size = 3.7 × 3.7 ${\mathrm{mm}}^{2}$, slice thickness = 5.0 mm) was used and the prediction filter, to mitigate the gating latency, was enabled. For drift motion tracking, a 0.5 Hz (0.25 Hz per imaging plane) T2‐weighted turbo spin echo (acquired pixel size = 2.0 × 2.0 ${\mathrm{mm}}^{2}$, slice thickness = 5.0 mm) was used.

### 2.4 Measurements

The geometrical and dosimetric E2E accuracy of the workflows was evaluated using three reference dose distributions for different scenarios. All scenarios were measured once, on the same day, using the same clinical MR‐linac to minimize differences caused by calibration or setup variations.

First, a static setup was evaluated as a benchmark scenario, in which no CMM was applied. This served as a benchmark for the dosimetric and geometric E2E accuracy of the workflow, and the results were expected to align with those of Oolbekkink et al.^9^

Following the static scenario, two scenarios without motion management (referred to as unmanaged scenarios) were performed with two types of motion: A $\cos^{4}$ motion ($A_{p2p}$ = 15 mm, 12 bpm) was used to simulate free breathing without motion management, and a linear drift of 2 mm/min was used to simulate drift motion. During these scenarios, motion was present during dose delivery but not managed in any form and are referred to as the unmanaged respiratory scenario, and unmanaged drift motion scenario, respectively.

Next, managed respiratory beam gating using CMM was assessed. The same $\cos^{4}$ motion was applied for phantom movement, and beam gating was performed using a gating envelope corresponding to the PTV margin surrounding the GTV. If the overlapping volumes of the GTV and the gating envelope fell below 95%, the beam was automatically turned off. This allowed for some motion within the gating window, but considerably less compared to the unmanaged scenario. The linac duty cycle during this measurement is approximately 71%.

Lastly, a linear drift of 2 mm/min was used to induce IDCs. When the beam was interrupted by CMM, an IDC was performed. The IDC was triggered when the overlap between the GTV and the gating envelope fell below 95%, corresponding to approximately 6.5 mm of drift relative to the reference position. At this point, the beam was automatically turned off. During IDC, the phantom motion was paused to prevent the motion platform from exceeding its maximum extension. Once the IDC calculation was completed and the plan was ready for delivery, the phantom motion was resumed.

### 2.5 Film dosimetry

During the experiments, EBT4 GafChromic (Ashland, New Jersey, USA) film dosimeters (lot 12182301) were used to measure dose distributions due to their high spatial resolution, which makes film dosimeters ideal for detecting motion‐induced variations in dose distributions.^19^ The film was fixed in the film cassette, and before performing the measurements, water was used to fill the small gaps between the cassette and the film to minimize the effect of air pockets.^9^, ^20^ To convert the optical density to absorbed dose, calibration films were irradiated immediately after the measurements under reference conditions with known doses.^21^, ^22^ Both the film measurements and calibration films were scanned at least 24 h after dose delivery. The optical density of the film measurements was then converted into absolute dose using in‐house‐developed software.

### 2.6 Analysis

To compare the reference dose distributions with the measured dose distributions, the online MRI scans were registered to the film dosimeters using point‐based registration of the MR‐visible markers and the central positions of the cut‐outs in the film dosimeters, following the method described by Oolbekkink et al.^9^ This process yielded a transformation matrix, which was then applied to the reference dose distributions, registering the reference dose distributions to the measured dose distributions.

The dosimetric E2E accuracy of the workflows was assessed by comparing dose difference plots and a global gamma index analysis utilizing a 2%/2 mm criterion and a minimum dose threshold set at 10% of the maximum measured dose.^23^

The geometric E2E accuracy of the workflow was evaluated by fitting the registered reference dose distributions to the measured dose distribution to obtain the shifts in the *x*‐ and *y*‐directions, as well as the rotation in the *xy*‐plane (along the *z*‐axis). The method used to obtain the geometric accuracy followed the approach described by Oolbekkink et al. using the Hooke–Jeeves direct search optimization method.^9^, ^24^ Since the phantom motion occurs along the *y*‐axis, only shifts along this axis are expected. Therefore, 1D line profiles along the *y*‐axis of the registered dose distribution were plotted for visual validation. For completeness, shifts in both directions and the in‐plane rotation were included in the analysis.

## 3 RESULTS

### 3.1 Dosimetric accuracy

**FIGURE 3:**
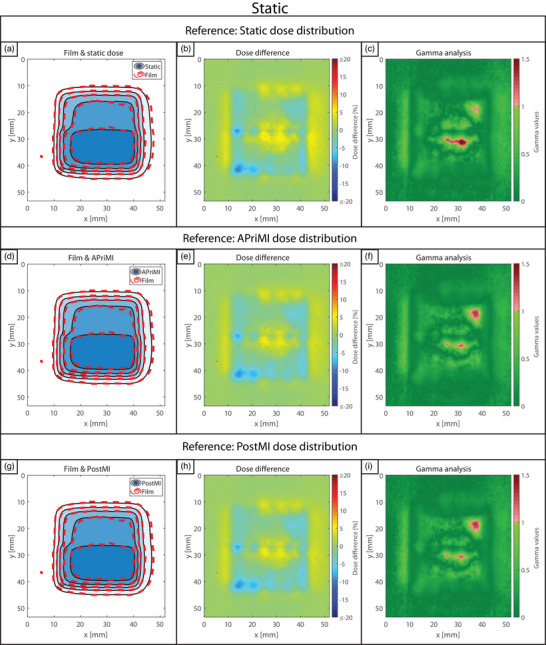
Dosimetric accuracy for the static scenario. (a), (b), and (c) show the isodose line agreement, the dose difference, and the gamma index analysis plot for the static dose distribution. (d), (e), and (f) show the isodose line agreement, the dose difference, and the gamma index analysis plot for the APriMI dose distribution, respectively. (g), (h), and (i) show the isodose line agreement, the dose difference, and the gamma index analysis plot for the PostMI distribution, respectively. The isodose lines represent dose levels of 2, 4, 6, 8, and 10 Gy. APriMI, A Priori Motion‐Included; PostMI, Posteriori Motion‐Included.

Figure 3 presents the static scenario, showing the static, APriMI, and PostMI dose distributions compared to the measured dose distribution. The isodose lines, dose difference, and gamma plots indicate highly comparable results for the static, APriMI, and PostMI dose distributions. This agreement between the three reference dose distributions and the measured dose distribution is also observed in the 1D dose profile (Figure 8a), and the gamma pass rates (Table 1).

**TABLE 1:** Gamma index pass rate for the measured dose distribution relative to the reference dose distributions, utilizing a criterion of a maximum DD of 2% and a DTA of 2 mm.

			Reference dose distributions		
Scenario	CMM used	Phantom motion	Static (%)	APriMI (%)	PostMI (%)
Static	No	None	98.5	98.8	98.8
Unmanaged	No	$\cos^{4}$	64.5	100.0	100.0
Unmanaged	No	Drift	17.8	N/A	100.0
Managed	Yes	$\cos^{4}$	76.7	98.6	98.5
Managed	Yes	Drift	46.7	N/A	95.5

Abbreviations: APriMI, A Priori Motion‐Included; CMM, Comprehensive Motion Management; DD, dose difference; DTA, distance‐to‐agreement; PostMI, Posteriori Motion‐Included.

**FIGURE 4:**
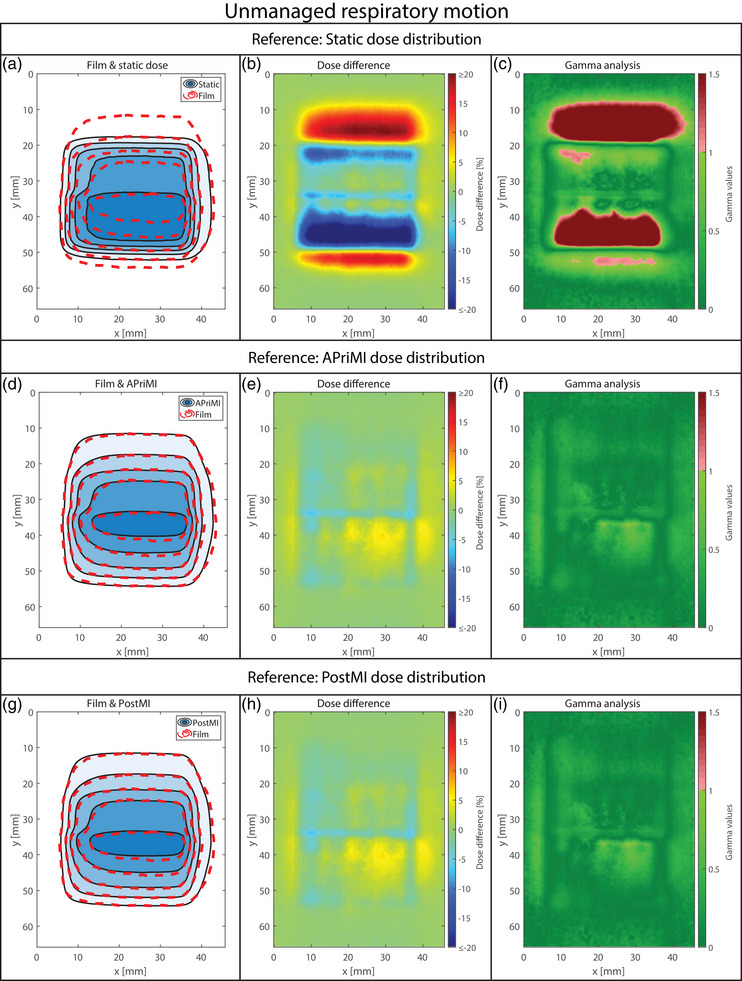
Dosimetric accuracy for the unmanaged respiratory motion. (a), (b), and (c) show the isodose line agreement, the dose difference, and the gamma index analysis plot for the static dose distribution. (d), (e), and (f) show the isodose line agreement, the dose difference, and the gamma index analysis plot for the APriMI dose distribution, respectively. (g), (h), and (i) show the isodose line agreement, the dose difference, and the gamma index analysis plot for the PostMI distribution, respectively. The isodose lines represent dose levels of 2, 4, 6, 8, and 10 Gy. APriMI, A Priori Motion‐Included; PostMI, Posteriori Motion‐Included.

**FIGURE 5:**
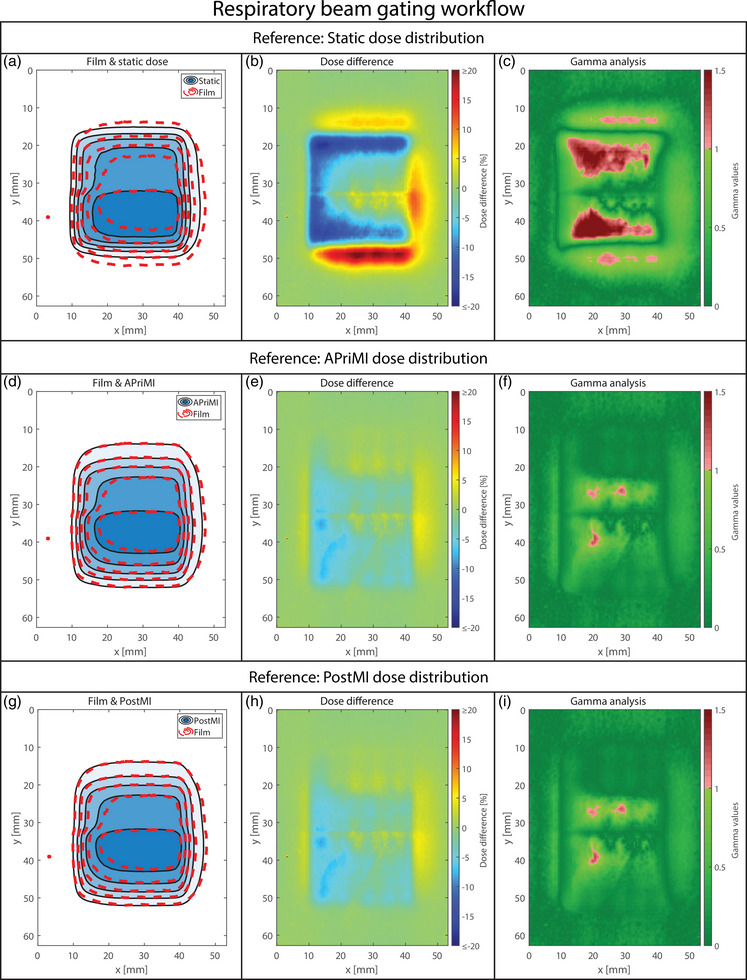
Dosimetric accuracy for the managed workflow of the respiratory motion. (a), (b), and (c) show the isodose line agreement, the dose difference, and the gamma index analysis plot for the static dose distribution. (d), (e), and (f) show the isodose line agreement, the dose difference, and the gamma index analysis plot for the APriMI dose distribution, respectively. (g), (h), and (i) show the isodose line agreement, the dose difference, and the gamma index analysis plot for the PostMI distribution, respectively. The isodose lines represent dose levels of 2, 4, 6, 8, and 10 Gy. APriMI, A Priori Motion‐Included; PostMI, Posteriori Motion‐Included.

**FIGURE 6:**
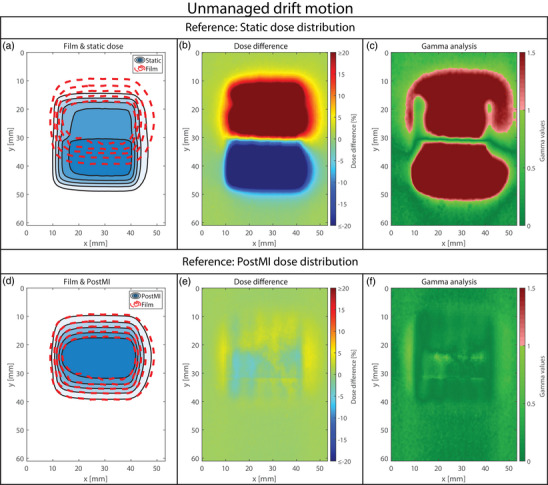
Dosimetric accuracy for the unmanaged linear drift motion. (a), (b), and (c) show the isodose line agreement, the dose difference, and the gamma index analysis plot for the static dose distribution. (d), (e), and (f) show the isodose line agreement, the dose difference, and the gamma index analysis plot for the PostMI distribution, respectively. The isodose lines represent dose levels of 2, 4, 6, 8, and 10 Gy. PostMI, Posteriori Motion‐Included.

**FIGURE 7:**
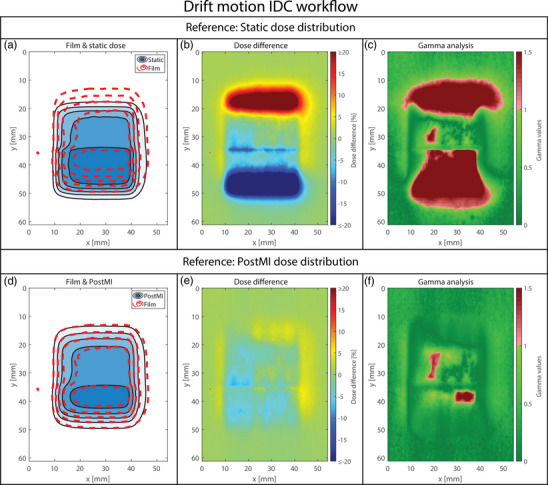
Dosimetric accuracy for the managed workflow of the linear drift motion. (a), (b), and (c) show the isodose line agreement, the dose difference, and the gamma index analysis plot for the static dose distribution. (d), (e), and (f) show the isodose line agreement, the dose difference, and the gamma index analysis plot for the PostMI distribution, respectively. The isodose lines represent dose levels of 2, 4, 6, 8, and 10 Gy. IDC, intra‐fraction drift correction. PostMI, Posteriori Motion‐Included.

**FIGURE 8:**
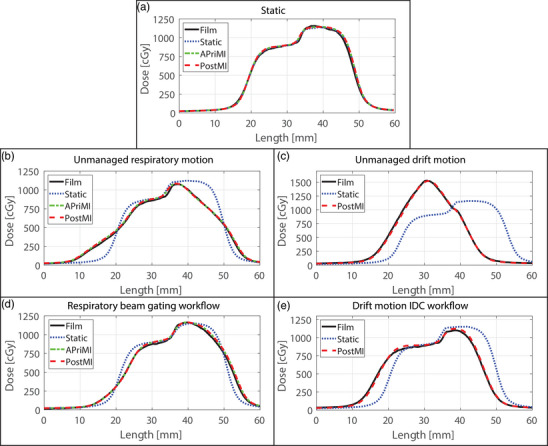
Registered *y*‐axis profiles of the measured and reference dose distributions for the static (a), unmanaged respiratory motion (b), unmanaged drift motion (c), the managed respiratory beam gating workflow (d), and the managed drift motion using the IDC workflow (e). IDC, intra‐fraction drift correction. APriMI, A Priori Motion‐Included; PostMI, Posteriori Motion‐Included.

The results of the dosimetric analysis for two respiratory motion scenarios are shown in Figures 4 (unmanaged) and 5 (managed). Results for the workflows involving drift motion are shown in Figure 6 (unmanaged) and 7 (managed). In Figures 4, 5, 6, 7, large differences are observed between the measured dose distributions and the static dose distributions, with gamma pass rates as low as 17.8%. Both the APriMI (where applicable) and PostMI dose distributions show excellent agreement with the measured dose distribution. This is further supported by the gamma pass rates (shown in Table 1), with the lowest gamma pass rate being 95.5%. These show again poor agreement for the static dose distribution and excellent agreement for both the APriMI and PostMI dose distributions relative to the measured dose.

### 3.2 Geometric accuracy

Table 2 presents the E2E geometrical accuracy of the workflows using the three reference dose distributions. Additionally, the 1D line profiles along the y‐axis are presented in Figure 8 for the: (a) static scenario, (b) unmanaged respiratory motion, (c) unmanaged drift motion, (d) managed respiratory motion, and (e) managed drift motion. Due to the large differences in dose distributions between the static dose and measurements, the fitting results in substantial geometric shifts along the *y*‐axis for the scenarios that involve drift motion, as expected. This is evident from Table 2 and Figure 8 and shows that the motion overshadows the workflow related uncertainties. Both the APriMI and PostMI dose distributions showed excellent agreement (for respiratory and drift motion) with the measured dose distributions, as observed by the 1D dose line profiles. Table 2 shows that the APriMI and PostMI methods produced results that align well with the static dose distribution, with similar geometrical shifts as obtained during the static benchmark scenario. As expected, the non‐moving axis showed only minor differences across all reference dose distributions, with the largest deviation being 0.8 mm for the static reference dose distribution during the motion‐managed scenario involving beam gating (see Table 2).

**TABLE 2:** Geometric accuracy for various scenarios evaluated using the static dose distribution, APriMI distribution, and PostMI distribution.

			Geometric accuracy								
			Static			APriMI			PostMI		
Scenario	CMM used	Phantom motion	*X* (mm)	*Y* (mm)	Rot. *Z* (deg)	*X* (mm)	*Y* (mm)	Rot. *Z* (deg)	*X* (mm)	*Y* (mm)	Rot. *Z* (deg)
Static	No	None	0.1	0.2	−0.3	0.0	0.2	−0.3	0.0	0.2	−0.3
Unmanaged	No	$\cos^{4}$	*0.2*	*0.5*	*0.2*	0.1	−0.2	−0.3	0.1	−0.2	−0.3
Unmanaged	No	Drift	*0.3*	*9.0*	*0.5*	N/A	N/A	N/A	0.0	0.1	−0.5
Managed	Yes	$\cos^{4}$	*0.8*	*−0.7*	*−0.1*	0.3	0.2	−0.2	0.3	0.2	−0.1
Managed	Yes	Drift	*0.2*	*3.5*	*0.3*	N/A	N/A	N/A	0.3	0.3	0.4

*Note*: Italicized numbers indicate shifts for moving scenarios with the static reference dose distribution.

Abbreviations: APriMI, A Priori Motion‐Included; CMM, Comprehensive Motion Management; PostMI, Posteriori Motion‐Included.

## 4 DISCUSSION

This study introduces a novel method for generating reference dose distributions for motion‐included E2E testing. When evaluating motion‐included intra‐fraction managed workflows, results show that without the inclusion of the known motion, large dosimetric and geometric deviations may arise, as demonstrated in this study, potentially hindering a proper evaluation of the workflows. By inclusion of the remaining motion within the gating window using the APriMI and PostMI dose distributions, excellent agreement with the measured dose was observed. These findings indicate that motion‐included reference dose distributions for E2E testing of intra‐fraction motion‐managed workflows enable the discrimination of motion and the workflow related uncertainties.

Both the geometrical and dosimetric E2E accuracy of the motion‐included workflows are high, and show comparable results to the static scenario consistent with previous findings in the literature.^9^, ^10^ Minor dosimetric differences were observed between both APriMI and PostMI dose distributions and the measured dose distribution. These differences can be attributed to uncertainties in the workflow itself (e.g., online image registration), but also the motion discretization, the film dosimeter, setup modeling, minor material inhomogeneities, and a research implementation of the GPUMCD algorithm that did not include the anterior body coil. However, the coil's influence is minimal, as evidenced by the static scenario where the static, APriMI, and PostMI dose profiles match closely (Tables 1 and 2).

Depending on the E2E test design, the APriMI dose distribution may be suitable for specific scenarios, while the PostMI dose distribution is universally applicable (see Figure 8). For the APriMI the applicability is determined by the motion characteristics during dose delivery and the dose delivered per segment. In this study, a high number of MUs per segment resulted in a long beam‐on time relative to the respiratory motion frequency, encompassing multiple complete respiratory cycles per segment. Consequently, the periodic motion was effectively incorporated into the dose distribution. Generally, treatment plans contain a large amount of segments with a small number of MUs per segment, increasing the uncertainty in the APriMI dose distribution, as it does not account for dose delivery occurring only during a part of the motion. For these types of treatment plans, the PostMI method can be employed to account for the lower MU delivery during part of the motion cycle. However, in this study to demonstrate the performance of the APriMI and PostMI techniques, a two segment treatment plan was generated to emphasize the dose deviations that can occur during motion. If an E2E test was performed with a clinical treatment plan featuring more segments, the motion‐induced differences might be less observable.

Evaluating unmanaged or managed drift motion when using the PostMI method, requires an externally triggered, time‐resolved detector to accurately estimate the motion‐included dose. Although one can attempt to estimate the beam's on/off times and duration, such estimates could inadvertently affect the motion‐included dose distribution, and thereby the E2E accuracy of the workflow. An external trigger that records time‐resolved data helps mitigate this uncertainty. Examples include an EPID panel, an ionization chamber, a scintillation detector, or, as used in this study, a logfile.

During this study, a limited set of motion parameters such as respiratory frequency, amplitude, and drift speed was evaluated to assess the proposed methods. Using these parameters, it was demonstrated that both the APriMI (when applicable) and PostMI methods can incorporate phantom motion into the reference dose file. With these methods, a suitable reference dose distribution can be generated. Future research aimed at evaluating motion managed workflows (e.g., CMM workflows on the Unity MR linac) should therefore focus on the influence of more complex motion patterns, including higher respiratory frequencies and patient derived respiratory motion.

Although motion‐managed workflows can be evaluated using a static dose distribution, substantial differences may arise if motion is allowed within the gating window, as demonstrated in this study. When a very small gating envelope is used (approaching a near‐static scenario), the static dose distribution may yield sufficiently accurate results. However, such conditions do not realistically reflect the clinical implementation of motion management workflows.

In this study, a phantom with a substantial ED inhomogeneity was used to intentionally reduce part of the dose in the high‐dose region, demonstrating how a moving anatomy affects the dose distribution. The limited presence of MR‐visible materials made automatic image registration challenging, so the MR‐visible markers in the film insert were used for manual adjustments. These corrections had minimal impact, as confirmed by the static scenario, which showed deviations similar to those reported by Oolbekkink et al. and Bernchou et al.^9^, ^10^ If a more clinically relevant setup is desired, such as an anthropomorphic thorax phantom with a (deformable) moving lung tumor and corresponding treatment plan, the methods presented here would still be applicable for generating a suitable reference dose distribution.

While the motion‐included reference dose distributions were used to assess the E2E accuracy of the workflow, they could also support motion‐included (retrospective) plan QA. This enables plan QA within a moving detector setup, integrating patient intra‐fraction motion into the QA process for motion‐managed workflows. To perform motion‐included QA, phantoms are needed that can be moved such as shown by for example Uijtewaal et al.^25^ Also, the time‐resolved scintillator points shown could be used as the external trigger required for the PostMI method.

Additionally, these methods allow for detailed investigation of the dosimetric effects of various breathing patterns. They can be used without measurements to identify conditions under which different motion management techniques may be less effective and help determine appropriate gating or margin parameters to meet the desired constraints. Beyond these applications, the predicted dose distributions could also be leveraged to develop more robust treatment plans, improve motion management strategies, or identify patients for whom standard motion management approaches may be insufficient. Furthermore, the E2E testing methods presented here were performed on a 1.5 T MR‐linac but could also be applied to other MR‐linacs, or linacs equipped with a motion tracking system.

## 5 CONCLUSION

In this study, it is demonstrated that a suitable reference dose distribution is essential for intra‐fraction motion‐included E2E testing for motion‐managed workflows. Our methods generate reference dose distributions that account for the effect of a moving anatomy on the delivered dose distribution, enabling E2E testing of motion‐included workflows. Using these methods, the effects of prediction algorithms, latencies, and IDC replanning on the delivered dose distribution can be evaluated.

In this work, a limited set of motion parameters, such as respiratory frequency, amplitude, and drift speed, was investigated to evaluate the methodology. Future research should extend this approach by incorporating patient‐derived motion patterns and systematically varying motion characteristics to further assess the E2E accuracy of gated and IDC workflows using motion‐included reference dose distributions as presented here.

## CONFLICT OF INTEREST STATEMENT

The authors declare no conflicts of interest.
